# Narrow Versus Broad Phenotype Definitions Affect Genetic Analysis of Language More Than Other Broad Autism Phenotype Traits

**DOI:** 10.21203/rs.3.rs-5457750/v1

**Published:** 2025-05-06

**Authors:** Jinchuan Xing, Mudassir Lodi, Judy Flax, Christine Gwin, Sherri Wilson, Amber Robinson, Steven Buyske, Linda Brzustowicz, Chris Bartlett

**Affiliations:** Rutgers, the State University of New Jersey; Rutgers University; Rutgers, the State University of New Jersey; Rutgers, the State University of New Jersey; Ohio State University ex

**Keywords:** Autism, Genetics, Family-based, Linkage Analysis, Reading Impairment, Language Impairment, DSM-5, Posterior Probability of Linkage

## Abstract

Autism spectrum disorder is a heritable neurodevelopmental condition that displays heterogeneity in both presentation and etiology and it often presents with concomitant communication difficulties. The hypothesis behind the New Jersey Language and Autism Genetic Study is that genetic heterogeneity for component phenotypes of autism spectrum disorder may be reduced relative to the disorder as a whole. We previously published an initial phase of this study with family recruitment that used very restricted inclusion/exclusion criteria for both autism and language deficits in other family members. Here we present an expanded sample that includes a wider range of phenotypic presentations in the autism and language domains. We found that our previous findings on 15q and 16q, connecting autism spectrum disorder and oral/written communication, are only relevant to the narrow autism spectrum disorder and language impairment phenotypes (with posterior probability of linkage of 57% and 33%, respectively) though addition of families did reduce both critical regions. After variant prioritization and additional filtration based on segregation patterns and functional annotations, we determined a set of 10 and 6 top candidate risk genes with strong association to language impairment and reading impairment, respectively. The top candidate genes include both genes previously implicated in neurodevelopmental disorders (e.g., *ZNF774* and *DNAH3*) and genes not previously reported but with strong evidence of being involved in neurodevelopmental phenotypes.

## INTRODUCTION

Autism spectrum disorder (ASD) is a neurodevelopmental condition frequently seen in clinical practice and defined in the Diagnostic and Statistical Manual of Mental Disorders (DSM-5) by impairment in social interactions along with evidence of either restricted interests or repetitive behaviors (American Psychiatric Association 2000). While progress on the genetics of ASD advances (Arnett, Trinh, and Bernier 2019; Woodbury-Smith and Scherer 2018), much of the underlying genetic architecture remains either undiscovered or unelucidated. Further, the genetic relationship of ASD to other developmental disorders and traits poses a particular challenge.

Sometimes endophenotypes are thought of as subcomponents of the larger ASD diagnosis and other times endophenotype analysis implies more of a comorbid trait model. To address the challenge of how candidate phenotypes genetically intersect with ASD, two general strategies are commonly used. On one hand, a simple additive comorbidity model implies ubiquitously common underlying genetics that may drive observed comorbidities. On the other hand, when a trait commonly occurs in only subsets of the ASD population, then that trait demarcates heterogeneity in contrast to a simple additive model. The latter heterogeneity model has been emphasized in ASD genetics research. Aspects of language ability (Bradford et al. 2001; Eicher and Gruen 2015; Spence et al. 2006), traits related to the broader autism phenotype (Gerdts et al. 2013; Piven et al. 2013; Woodbury-Smith et al. 2018; Woodbury-Smith and Scherer 2018), and other cognitive traits (Clarke et al. 2016) have been applied to the genetic analysis of ASD.

The New Jersey Language-Autism Genetics Study (NJLAGS) took a different approach to study the genetics of communication in ASD (Bartlett et al. 2012; Bartlett et al. 2014). The goal of the project was to characterize the genetic relationship between communication skills and ASD. Families were ascertained only if they had both ASD and psychometrically verified language impairment (LI) within a candidate family. The co-incident diagnoses within the family presumed a shared genetics model between the two disorders. The genetic analysis model for NJLAGS also assumed at least partial common etiology (Bartlett et al. 2014), in order to focus on genetic loci that are common between the two disorders while attenuating any loci unique to each disorder. In our analysis, loci were found on chromosomes 15q23–26 (spoken language and ASD) and 16p12 (written language and ASD). Additional analyses indicated that the best model fit on how spoken language and ASD are related at chromosome 15 was the etiological equivalence model. On chromosome 16 the model fit for written language and ASD was equivocal, though the evidence for linkage was not as strong at that locus and the etiological equivalence model was not ruled out.

An additional key feature of NJLAGS goes beyond the co-incident diagnoses of language impairment and ASD within families. In the first wave of NJLAGS recruitment (WAVE1), proband criteria were conservative for both ASD and LI. ASD probands were required to meet the strict congruence of both DSM-IV Autistic Disorder (AD) and Autism Diagnostics Interview-Revised (ADI-R)/Autism Diagnostic Observation Scales (ADOS) criteria while LI probands met criteria for a definition of Specific Language Impairment (SLI) from the language disorders literature requiring typical intelligence quotient along with traditional criteria for language impairment (Bartlett et al. 2004; Bartlett et al. 2002). Coinciding with the publication of DSM-5 (American Psychiatric Association 2013) and out of concern that the narrow definitions of AD during WAVE1 of NJLAGS recruitment might may not generalize to the wider population of ASD families, ASD ascertainment criteria were relaxed in the second wave of recruitment (WAVE2) to conform with the new, more inclusive definition of ASD. Our rationale was that less strict criteria would increase ecological validity of genetic findings. Specifically, during WAVE2 recruitment, the families were ascertained using DSM-5 criteria which meant that those probands who had significant social impairments as well as evidence of restricted interests and repetitive behaviors, but did not meet the AD cutoff, were classified as ASD. During WAVE2 recruitment the LI criteria remained substantially the same although the IQ requirement was not taken into consideration and the term Developmental Language Disorder was adapted to explain greater inclusivity. See the Materials and Methods section for specific diagnostic criteria.

In this study, we identified candidate genes within the two linkage regions of interest, 15q23–26 and 16p12 originally defined in WAVE1 and further examined with WAVE2. The top candidate genes in the linkage regions indicate a shared etiology of ASD and language impairment at these two loci. We also present the results of a unified analysis of genetic heterogeneity across both waves of recruitment (WAVE1 and WAVE2) to better characterize our previous genetic findings and to assess if candidate genes generalize across narrow versus broad phenotypic criteria-based ascertainment schemes. If strict ASD and LI are the same as those disorders under more relaxed criteria, then that genetic homogeneity should drive up the previous linkage peaks and the candidate genes should be the same. The same reasoning extends to analysis of broader autism phenotype traits. As part of NJLAGS and consistent with ASD criteria, we also collected component phenotypes for the social and restricted interests/stereotyped behavioral domains. These phenotypes, while secondary to the goal of NJLAGS, nevertheless offer insight for understanding heterogeneity in ASD and the relations among related neuropsychiatric disorders. If strict ASD (AD diagnosis) is not genetic etiologically different than ASD under more relaxed criteria, linkage results should be more congruent across the two phases of the study.

## MATERIALS AND METHODS

### I. Families

#### Design of study.

The purpose of the NJLAGS study is to find genetic variation relevant to both ASD and spoken language impairments (Bartlett et al. 2012; Bartlett et al. 2014). The goal of recruiting families was to find close relatives where at least one person had ASD and at least one other person had a language impairment. The entire family was then enrolled in the study including extended family members, when available, raising the prospects of finding additional persons with ASD and/or LI. Any genetic factors that “reduce” language ability in the person with LI should also do so in persons with ASD, and possibly to greater effect through gene-by-gene or other interactions as evidenced and discussed elsewhere (Bartlett et al. 2012). All subjects gave informed consent or assent conforming to the guidelines for treatment of human subjects supervised by the Intuitional Review Board at Rutgers, The State University of New Jersey (IRB number: 13–112Mc).

While the NJLAGS study manifestly focused on communication in autism, the genotype-phenotype relationship among all family members was considered highly relevant. Therefore, all family members—including higher-functioning persons with ASD—received the age-appropriate version of language, reading, and other cognitive and behavioral measures used to define ascertainment criteria and phenotypes for genetic analysis. Questionnaire data related to other behaviors associated with ASD and communication were collected for all subjects.

#### Ascertainment in two waves of recruitment.

In our previous study sample (WAVE1) (Bartlett et al. 2014), we defined tiers of analysis that ranked from strict phenotypically narrow (Tier I) to phenotypically broader (Tier II). Tier I families included at least one autism proband who met criteria for DSM-IV Autistic Disorder (AD) and at least one proband who met strict criteria for Specific Language Impairment (SLI). Tier II families were multiplex for AD (including one proband with LI) but with no family member with SLI. (We note also that in the 2014 publication there was a Tier III that consisted of trios and a non-European family which we do not carry through to the analysis presented here.)

Our goal during WAVE2 was to assess the ecological validity of the relationship between ASD and communication impairments by loosening phenotypic criteria, which would better represent the larger population of ASD families. This was done by 1) using the more inclusive DSM-5 diagnosis of ASD and 2) using the less restrictive language impairment criteria for SLI, which excluded the normal IQ requirement and is more aligned with the current definition of Developmental Language Disorder (DLD) or LI.

#### Hypothesis.

We anticipated that we would find more heterogeneity with regards to language phenotypes because our WAVE2 families included some families who were multiplex for ASD with and without concomitant LI, some with the previous, more stringent AD/SLI diagnoses, and some with the new ASD/DLD (LI) diagnoses. The effect of WAVE2 on social quantitative traits would be anticipated to introduce minimal heterogeneity since the Social Responsiveness Scale-2 (SRS-2), for example, is a valid metric across the full phenotypic range of scores seen in the population, including scores associated with autism.

#### Characteristics of the families.

The first phase of ascertainment (WAVE1) included 440 individuals from the 79 families. In WAVE1, we used an autism proband criteria whereby two of three diagnostic criteria were met: 1) Autism Diagnostic Interview – Revised (Lord, Rutter, and Le Couteur 1994) (ADI-R) algorithm scores meet cutoff for “autism,” 2) Autism Diagnostic Observation Schedule (Lord et al. 2000; Lord et al. 1989) (ADOS) algorithm scores meet cutoff for “autism,” 3) DSM-IV (American Psychiatric Association 2000), autistic disorder. We also sought to ascertain language impaired probands in the same family using the following criteria: 1) A core standard score of <= 85 on the age appropriate version of the Clinical Evaluation of Language Fundamentals (Semel, Wiig, and Secord 2003; Wiig, Secord, and Semel 2004) (CELF-4 or CELF-Preschool) or at least one standard deviation below peers on 60% of the administered oral language subtests as well as a significant history of language-learning intervention. The last criterion is based on our past research. Historical information on past language interventions has been useful to identify normalized adults that had language impairment in their youth but did not meet our defined threshold for language impairment upon testing in adulthood although many present with concomitant language based reading disorders as adults (Flax et al. 2003; Tallal et al. 2001). 2) Performance IQ (PIQ) >= 80 on the Wechsler Abbreviated Scale for Intelligence (WASI) (Wechsler 1999), 3) Hearing within normal limits during a traditional audiological screening, 4) No motor impairments or oral structural deviations affecting speech or non-speech movement of the articulators, 5) No history of ASD or frank neurological disorders such as intellectual disability or brain injury, as determined by parental interview, 6) Native English speaker with English as the primary language spoken at home.

#### Phenotypic Modifications in WAVE2.

The WAVE2 samples included 192 individuals from 36 families. We used broader proband criteria based on the DSM-5 ASD, which included but was not limited to AD. We also sought to ascertain at least one language impaired proband in the same family using the same criteria as in WAVE1 without the PIQ >=80 requirement. Additionally, we accepted multiplex ASD families into the study as long as at least one of the ASD probands had a concomitant oral language impairment (and if there were no LI proband elsewhere in the family, we accepted multiplex ASD families as long as both were language impaired).

Once a family was ascertained and enrolled in the WAVE2 part of the study, we applied phenotypic definitions described below for linkage analysis. The ascertainment criteria for probands outlined above was only for ascertainment purposes. For genetic analysis, a series of traits were derived to illuminate key aspects of how ASD relates to language and broader autism traits within families.

#### DSM-IV and DSM-5 Criteria for ASD.

The previous NJLAGS studies (Bartlett et al. 2012; Bartlett et al. 2014) included only DSM-IV (American Psychiatric Association 2000) criteria for both ascertainment and analysis. Since the DSM-5 (American Psychiatric Association 2013) was published, it has been widely adopted in the current scientific literature; we applied the DSM-5 criteria to all new families in our study (retroactively for families ascertained prior to DSM-5) in order to have a uniform definition of affected with ASD for downstream analysis. This has the effect of changing classifications for some participants and thereby potentially including or excluding families compared with our previous research. Individuals from WAVE1 who were not considered Autism probands because they did not meet the strict cutoffs were now reclassified as ASD and included in the current analyses with their new diagnoses (N=3). While we expected that such phenotypic change would induce differences from our previous results, results from this paper will more easily align with current and future autism genetics research in the literature.

#### Language Phenotypes.

Two categorical phenotypes covered the range of phenotypic variably of interest from our previous studies (Bartlett et al. 2004; Bartlett et al. 2002; Simmons et al. 2010): 1) Language impairment, 2) Reading Impairment. Language impairment or “LI” referred to the LI requirements described previously. We defined our language impairment trait LI* that included all persons with ASD as affected, along with any person without ASD that met the LI criteria. This is an etiological equivalence phenotype model described elsewhere (Bartlett et al. 2014), for the purpose of finding loci relevant to both LI and ASD.

A determination of reading impairment (RI) required scores at least 1 standard deviation (SD) below the population mean on 60% of all reading tests and subtests. As with LI*, we defined a parallel RI* that included all persons with ASD as affected, along with any person without ASD that met the RI criteria. While RI is overtly defined by performance on written language tests, we include it since in our previous studies of multiplex language impairment families (without ASD) we observed many instances of semi-compensated adults with a historical childhood diagnosis of language problems and currently demonstrating weak oral language skills who did not qualify for language impairment but did meet reading impairment criteria. Use of RI has improved gene mapping in language impaired families that have such semi-compensated adults (Bartlett et al. 2004; Simmons et al. 2010).

#### Non-language Phenotypes.

The test battery also included representative metrics from the broader autism phenotype. For the social domain we administered the SRS-2, a commonly used instrument to assess social functioning in the ASD spectrum that is also valid for detection of variation in the general population (Constantino et al. 2004; Constantino et al. 2003; Constantino, Hudziak, and Todd 2003). The SRS was administered and analyzed on all participants. The SRS Total T-score was of main interest due to its wide deployment in the field of ASD research as a general metric social functioning. The most current version (SRS-2) contains five socially motivated subscales, one of them being Restricted Interests and Repetitive Behaviors (RIRB). During WAVE1 the SRS raw scores were analyzed as quantitative traits and as categorical traits where we chose the mild-moderate cut-off for affection (Bartlett et al. 2014). We did not analyze the scores from the RIRB subscale separately since they were not available for adults during our analysis period. Since we now were able to use both quantitative and categorical scores from the SRS-2 for an overall Social functioning score (Total T-score) as well as information from the RIRB subscale, we elected to define our RIRB phenotype using the SRS-2 in place of the Yale-Brown Obsessive-Compulsive Scale (Y-BOCS, CY-BOS, C-YBOCS ASD) (Scahill et al. 2006; Goodman, Price, Rasmussen, Mazure, Fleischmann, et al. 1989; Goodman, Price, Rasmussen, Mazure, Delgado, et al.1989), which was used in our earlier work.

### II. Genotyping

Genome-wide data was obtained through single-nucleotide polymorphism (SNP) array genotyping conducted in rolling waves as families were ascertained. The WAVE1 sample SNP data was generated using the Affymetrix Axiom 1.0 array (Affymetrix, Santa Clara, CA), which included 567,893 SNPs and lower frequency variants. WAVE2 sample SNP data was generated with the Illumina Infinium PsychArray-24 v1 array (Illumina, San Diego, CA), which includes 593,260 SNPs and lower frequency variants. In this study, we focused on SNP data (population minor allele frequency >1%). Quality control on these SNP genotypes was conducted by array batch and by array type, as described previously (Simmons et al. 2010), including with regard to individual/SNP genotype completion, relationship checking, Mendelian errors, and ancestry. Only samples that clustered with the CEU samples from HapMap reference data, as determined by EIGENSTRAT using the recommended parameters in the documentation (Price et al. 2006), were included in the linkage analysis. For linkage analysis, a subset of 10,899 SNPs in common across both array types was chosen that minimized marker-to-marker linkage disequilibrium while retaining a high frequency of minor alleles (>30%) so as to provide suitable genomic coverage of recombination events in the pedigrees. This map was augmented with an additional 5,325 SNPs that were not in common across the datasets, mostly near the ends of the chromosomes, to increase information content as assessed by MERLIN 1.1.2. Within each family, the same array was used for genotyping so that adding SNPs to increase information content did not induce a pattern of missing data within families.

### III. Statistical Analysis

Linkage analyses were conducted with KELVIN 2.3.3 (kelvin.mathmed.org). KELVIN implements the posterior probability of linkage (PPL) metric to estimate the probability that a genetic location is linked with a tested trait (Vieland et al. 2011). Genetic map locations came from version 3 of the Rutgers Combined Physical-Linkage Map project (Matise et al. 2007; Kong et al. 2004). All centiMorgan (cM) values were reported as sex-averaged Kosambi map units.

Primary linkage analysis of the two language categorical traits characterizes the genetic relationship between ASD and language (oral and written). Secondary analysis of the broader autism phenotype was also conducted on both quantitative and categorical traits derived from the same scales (the latter based on a threshold). These secondary analyses include information across the range of values seen in the general population as well as in ASD on a single scale.

#### Stratification to assess heterogeneity.

Our chosen Bayesian analysis method allowed us to address the question of postulated genetic heterogeneity by analyzing the data stratified on phenotypic criteria. The two waves and two tiers stratified the families for analysis, while the phenotypic definitions outlined above were used to categorically define individuals for analysis. Every phenotype was analyzed twice. Our chosen statistical metric came from a Bayesian analysis where the posterior probability of linkage is sequentially updated across the phenotypic subgroups of the data to output a single PPL at each locus. We present this single output as the primary linkage statistic of interest to evaluate the strength of the evidence at a given location. In practice, this procedure is performed across the four subgroups formed by the two recruitment waves of the study and the two tiers within each phase (2 Waves x 2 Tiers = 4 phenotypic subsets). The main advantage of the Bayesian sequential updating over a single pooled analysis of all the data is that a valid linkage signal in a subset of the data is less attenuated (Huang and Vieland 2001), and hence more likely to be detected. Importantly, retaining valid linkage signals within a subset using Bayesian updating is accomplished without causing an inflation in the test statistic since we are not maximizing over many separate analyses (Vieland 1998). Once linkage is established, a subsequent *post hoc* inspection of each constituent dataset provides information on the structure of genetic heterogeneity at a linked locus. A secondary linkage analysis was conducted using all families jointly in a single ‘pooled’ analysis of each trait. By comparing the sequentially updated result to the pooled result, we may qualitatively infer the role of heterogeneity in the dataset. Since stratifying on an *irrelevant* sample characteristic will, on average, produce the only slightly lower PPL results compared to a pooled analysis (Govil and Vieland 2008), if we observe appreciably higher sequentially updated PPL over pooled PPL results we may infer that heterogeneity demarcated by the stratifying variable is present in the data.

The PPL is an estimated probability, in the Bayesian sense, though researchers may wish for a guide in terms of the more commonly used and familiar p-value. In order to assess the effect of performing genome scans using multiple correlated phenotypic traits, we have performed simulation studies on 2,500 unlinked genomes in the NJLAGS family collection (genotypes generated without regard to phenotypes) and analyzed each to create an empirical null distribution for estimating p-values (Bartlett et al. 2014). A PPL of 0.32 or greater is consistent with a genome-wide error rate of p<0.001, a PPL of 0.26 corresponds to p<0.01, and a PPL of 0.11 corresponds to p<0.05.

### IV. Association Analysis

Under the linkage peaks we performed posterior probability of linkage disequilibrium (PPLD) analysis (Huang and Vieland 2010), the association analog of the PPL for family data implemented in KELVIN 2.3.3 (Vieland et al. 2011). We analyzed genotypes imputed from the array-based data on the Haplotype Reference Consortium (http://www.haplotype-reference-consortium.org/site) (McCarthy et al. 2016) using an r^2^ threshold of 0.3. Post-imputation we kept N=116,675 SNPs in the linkage regions with minor allele frequency (MAF) ³5%, imputation quality score ³0.4, and Hardy-Weinberg equilibrium p-values ³1×10^−5^. Critical region for linkage analyses were defined, as previously described (Bartlett et al. 2014), from the maximum PPL in both directions along the chromosome from the peak until a PPL of <5% is reached. All SNPs within these regions were analyzed for association with the phenotype appropriate for that critical region.

### V. Candidate Gene Analysis

#### a. Single Nucleotide Variant (SNV) and insertion/deletion (indel)

##### SNV/indel Call Set.

The SNV/indel call sets were obtained from a previous study (Zhou et al. 2023). Briefly, whole-genome sequencing (WGS) was conducted using Illumina sequencing platform and the variant calling was performed using the GATK v3.5.0 variant calling pipeline following the best practice recommendation (DePristo et al. 2011).

##### SNV/indel Analysis and Candidate Gene Identification.

pVAAST (pedigree Variant Annotation, Analysis, and Search Tool) (Hu et al. 2014) was used for candidate gene prioritization within the LI* and RI* linkage regions, respectively. Variants were filtered to include those that have an MAF < 5% in the ExAC dataset. For the control dataset, 635 GTEx WGS samples were obtained from the GTEx project and condensed into a single group (Zhou et al. 2023). pVAAST (Hu et al. 2014) was run under both autosomal dominant and autosomal recessive models. Under the autosomal dominant model, all family members (including the proband) descended from one pair of ancestors were analyzed. Under the autosomal recessive model, only the parents and siblings of the proband are incorporated in the analysis. As such, multiple sub-pedigrees were created for some families. In these cases, the sub-pedigree with the most affected members sequenced was selected for the final analysis. In the case of multiple sub-pedigrees having the same number of affected members sequenced, the sub-pedigree with the most family members sequenced was selected. pVAAST calculates a pVAAST score using variant linkage pattern, association evidence, MAF, and functional prediction. The significance of each gene was determined by 10^6^ permutations per gene. The pVAAST command used is as follows:

VAAST−mpvaast−o{output_file_path}−pv_control{ctl_file}−regionLI.bed{gff_file}{cdr_file}−gw1e6−min_suc50−p22−max_time300

To select genes for further study, a p-value threshold was used for each of the two phenotypes. The p-value threshold corrected for multiple comparison is calculated by dividing 0.05 by the total number of genes in the region.


$$\text{LI}*:\;0.05\;/\;308\;=\;1.62{\text{e}}^{-4}$$

$$\text{RI}*:\;0.05\;/\;103\;=\;4.85{\text{e}}^{-4}$$

#### a. Structural Variant (SV) and Copy Number Variant (CNV)

##### Candidate SVs and CNVs.

The candidate SVs and CNVs were obtained from a previous study (Alibutud et al. 2023). For the SV call set, SVs were generated based on the WGS data from 272 individuals across 73 families using MetaSV (Mohiyuddin et al. 2015). The mobile element insertions (MEIs) were identified using MELT (Gardner et al. 2017). The SV set and MEI set was then merged and annotated using AnnotSV (Geoffroy et al. 2018) for functional prediction. GEMINI (Paila et al. 2013) is then used to determine the inheritance patterns (Alibutud et al. 2023). Variants that were identified as benign by AnnotSV were filtered out. The final candidate SV set consisted of 1,816 variants and 739 genes (Alibutud et al. 2023).

For the CNV call set, samples were genotyped using either Affymetrix Axiom 1.0 Genome-Wide CEU 1 (Affymetrix, CA, USA) or Illumina Infinium PsychArray (Illumina, CA, USA) (See Genotyping section above for details). Two CNV calling algorithms were applied to determine the call set: PennCNV (Wang et al. 2007) and QuantiSNP (Colella et al. 2007). CNVs were then filtered to remove CNVs that were too small (<10 kb) or too large (>7.5 Mb). Additionally, CNVs that were called by less than 5 probes were filtered out. In the final CNV set, there were 2528 variants across 524 individuals.

Using this final set of candidate SVs and CNVs, variants within the linkage regions of interest were selected. The genes affected by the SVs and CNVs were used in the subsequent analyses.

#### b. Candidate Gene Filtration and Prioritization

##### Initial Filtration.

The autosomal dominant and autosomal recessive gene sets from the SNV/indel analysis were combined with the prioritized gene sets from the CNV and SV analyses to obtain a final set of candidate genes. Genes were then annotated for brain expression using three databases, GTEx (Aguet et al. 2019), BrainSpan (Miller et al. 2014), and Human Developmental Brain Resource (Lindsay et al. 2016). Each database contains median transcripts per kilobase million (TPM) values for brain tissues or developmental stages. The max brain TPM value across each database was considered in the final analysis for each gene. A cutoff of max TPM value > 5 was used to select candidate genes. Genes were also annotated for predicted constraint metrics using gnomAD (Collins et al. 2020). Metrics considered in the analysis include pLI score (probability of being loss of function), O/E LoF score (observed/expected loss of function), LOEUF (loss-of-function observed/expected upper bound fraction), and missense Z-score.

##### Segregation Analysis.

Pedigree data and genotype information from pVAAST and SV/CNV results was then analyzed to determine the variant segregation pattern. Specifically, variants were filtered based on LOD score per family, with a LOD score threshold of > 0.3. Variants segregating in at least one family after LOD score filtration were considered for the final candidate gene set. In addition to the set of families containing each variant type for each gene, a set of unique families for each gene was determined. This set of unique families represents all families that contain a variant within a given gene, regardless of variant type. Lastly, genes were annotated based on previous association with a neurodevelopmental disorder (NDD) (Zhou et al. 2023).

##### Top Candidate Gene Selection.

Selection of top candidate genes from the combined annotated set was then performed using the following criteria: 1) a gene contains variants that segregates in multiple families 2) a gene has brain expression max TPM value > 5. Genes were ranked higher if they possessed a max TPM value > 5 in multiple databases. Additionally, gene association with other NDDs was considered.

### VI. Data Availability

The raw sequencing reads, variants, and genotypes for all samples are available in the National Institute of Mental Health (NIMH) Data Archive (NDA) under collections C1932 and C2933 and NRGR under study 39. Access can be requested through the portal at this link (https://nda.nih.gov).

## RESULTS

### I. Refining the Critical Regions on Chromosomes 15 & 16 for Language-Related Traits

Genome-wide multipoint linkage analysis results are visualized in Figure 1 and specific aspects of peaks are detailed in Table 1. Figure 1 shows the primary analysis results that we use to declare linkage to a locus, which is based on Bayesian sequential updating across phenotypic subsets. The LI* phenotype on chromosome 15 (PPL=57%) has a similar magnitude as the previous study (i.e., no change from Bartlett et al. 2014) and remains genome-wide significant with a slightly smaller critical region (0.4 Mb reduction). RI* on chromosome 16 (PPL=33%) likewise, showed the same trend with a similar magnitude (PPL reduced 3%) from the previous study, though with a smaller critical region (1.6 Mb reduction).

No new (significant) peaks were noted for LI* or RI* and no new suggestive peaks were observed. The nominal (p<.05) RI* peaks on chromosomes 1 and 5 are notable only since both were also observed in the WAVE1 study, and in both cases those peaks have a larger magnitude when analyzed together with WAVE2. Chromosome 1 went from 9% to 14%, and chromosome 5 went from 16% to 19%.

#### Analysis of Heterogeneity.

Compared with the pooled results in Table 2 (in the column labeled “Pooled Tiers and Waves”), which shows PPL values for LI* and RI* in a pooled analysis of both WAVE1 and WAVE2 in a single analysis, both significant peaks on chromosomes 15 and 16, respectively, were attenuated. LI* remains significant with a PPL of 44%, though RI* is reduced to nominally linked status with a PPL of 23% (i.e., less than suggestive linkage). Under heterogeneity, sequentially updated linkage signals would be expected to be larger than a pooled analysis. Our observed decrease when adding the WAVE2 data is consistent with heterogeneity across data subsets. To assess differences between subsets we compared the PPL values for WAVE1 versus WAVE2. As suspected, linkage results only come from WAVE1 families. WAVE2 contributes nothing to LI* with a PPL of 2%, which is the prior probability of linkage, indicating neither evidence for nor against linkage. WAVE2 provides evidence against linkage of RI* to chromosome 16 with a PPL of 1.9%, which is less than the prior probability of linkage (2%) and therefore indicates evidence against linkage at that location.

We also performed an analysis with tiers pooled within wave, but with sequential updating across those pooled waves (in Table 2, the column labeled “Pooled Tiers-Sequentially Updated Waves”). Under this secondary analysis, decreases in the PPL from our primary analysis are indicative of heterogeneity between tiers. Table 2 shows only minor changes in the linkage signal of the language traits. Whereby LI* PPL goes from 57% to 61%, as noted above, the linkage signal comes entirely form WAVE1 and the original WAVE1 study indicated essentially homogeneity across the two tiers in terms of LI*. This pattern is mirrored in RI*, where the PPL was reduced from 33% to 32%, essentially the same value. Again, the linkage evidence comes entirely from WAVE1 and the previous study of WAVE1 indicated homogeneity across the two tiers in terms of RI* (Bartlett et al. 2014).

### II. Analysis of Non-language Traits

#### Social domain.

The SRS total score provides a quantitative overview of social reciprocity across the full continuum of behaviors that we used as a trait. Genome-wide analysis results are summarized in Figure 2. Analogous to the results from the language related traits, our primary analysis consisted of the sequentially updated PPL, updated over the data subsets for a single linkage metric. The largest peak was observed on 15q (PPL = 93%). This peak is not overlapping with the LI* locus and is unlikely to be related (Table 1). Chromosomes 2 (PPL = 41%), 3 (PPL = 34%), and 20 (PPL = 36%) also had significant peaks. All four of these peaks were present in the original WAVE1 analysis, but only the chromosome 15 peak was significant. We also note that the SRS-2 T scores used in the present analysis changed WAVE1 results relative to the raw scores used in the original publication. A suggestive peak on chromosome 19 (PPL = 27%) did not have even a nominal peak in the WAVE1 data.

Dichotomizing a quantitative trait transforms approximately continuous scores into a different phenotypic model that may provide different linkage information. In keeping with this assumption, analyzing the SRS as a dichotomous trait (SRS-DT) provided a new peak on chromosome 14 (PPL = 55%), as seen in Figure 2. A chromosome 15 peak was present at the same location as the quantitative trait but much diminished (PPL = 48%).

#### Heterogeneity in the Social Domain.

When compared with the pooled PPL runs (in Table 2, the column labeled “Pooled Tiers and Waves”), all SRS total score quantitative peaks were greatly diminished, though 15q stayed high at 80% (and significant though somewhat lessened) and chromosome 3 dropped from 34% to 26% (suggestive). These results suggest heterogeneity for all peaks, perhaps markedly so for the non-15q peaks. Inspection of the PPL results by subset bear this pattern out on chromosome 3 where WAVE2 provides evidence against linkage, and on chromosomes 19 and 20, where WAVE1 provides evidence against linkage. For the peaks on chromosomes 2 and 15 both WAVE1 and WAVE2 provide evidence for linkage (Table 2).

For the dichotomous trait, the pooled results have greatly diminished PPL results at the two peaks, and no new peaks were found. This pattern is consistent with heterogeneity. In this case, both WAVE1 and WAVE2 data are consistent with linkage at both loci, however, these linkage signals are clearly driven only by WAVE1 data (Table 2).

#### Restricted interests/repetitive behaviors.

The SRS also includes a quantitative subscale for restricted interests and repetitive behaviors (SRS-RIRB), the second diagnostic pillar that defines autism. The sequentially updated PPL summarized in Figure 2 shows two significant peaks, on chromosomes 3 (PPL = 71%) and 19 (PPL = 42%). Both peaks overlap with the SRS total score analyses, but are higher, suggesting that restricted interests and repetitive behaviors may be driving the SRS total score linkage results at those loci. When looking at the pooled results (Table 2, the column labeled “Pooled Tiers and Waves”), those same peaks are greatly attenuated from apparent heterogeneity. Indeed, when looking at the PPL’s for WAVE1 and 2 separately, WAVE2 shows evidence against linkage to chromosome 3, and is largely equivocal at chromosome 19.

### III. Candidate gene analysis

Initially, association analysis was conducted in each critical linkage region. No PPLDs warranted additional follow-up; where the largest PPLD was only 5%, well below any threshold for expending additional resources. Next, we identified candidate genes associated with LI* and RI* within the significant linkage regions. SNV, indel, and SV call sets based on the WGS data, as well as CNV call sets based on the genotyping array data are available for the NJLAGS cohort (Alibutud et al. 2023; Zhou et al. 2023). Using the genetic variants, we performed candidate gene prioritization using several criteria, such as variant impact, variant segregation pattern, gene function, and gene expression pattern in brain regions (see Methods for detail).

#### SNV/indel analysis.

The SNV/indel analysis was conducted for two modes of inheritance: autosomal dominant and autosomal recessive. As described in the Methods section, the autosomal dominant model analyzed all family members (including the proband) descended from one pair of ancestors. The autosomal recessive model analyzed only the parents and siblings of the proband. For LI*, 55 genes were scored under the dominant model, and 8 genes were scored under the recessive model (Table S1a and Table S1b). For RI*, 24 genes were scored under the dominant model, and 5 genes were scored under the recessive model (Table S1c and Table S1d). To determine the final candidate gene set for each phenotype, a union was performed between the dominant and recessive gene sets, and a p-value cutoff was applied (see Methods for more detail). After application of the p-value filtration, there were 29 genes in the LI* set, and 13 genes in the RI* set.

#### CNV analysis.

The initial sets of CNVs associated with language impairment and reading impairment were obtained from a previous study (Alibutud et al. 2023). Candidate CNVs were then filtered based on their location in the genome. After this filtration, there was one candidate CNV (15_90794757_90950358_<CN3>_1) located within the LI* linkage region and affects six genes. For RI*, two overlapping CNVs (16_21538186_21731120_<CN3>_1 and 16_21608472_21747738_<CN3>_1) were identified within the RI* linkage region, and both affect the same four genes. Given the two CNVs were identified in the same individual by different methods, it is likely that the two CNVs are one variant which different tools reported different break points. All three CNVs were *de novo* duplications (Table S2).

#### SV analysis.

Similar to the CNV analysis, the initial sets of SVs associated with LI* and RI* were obtained from a previous study (Alibutud et al. 2023). Candidate SVs within the LI* and RI* linkage regions were first identified, and benign variants based on AnnotSV annotations were filtered out. After this filtration, there were 21 candidate SVs identified for LI*, affecting 18 genes. There were 12 candidate SVs identified for RI*, affecting 11 genes (Table S3). The majority of the SVs are intronic, except for one deletion (15_79220669_79233894_DEL_1) and two insertions (15_77092501_77092758_INS_1, 16_20843131_20844439_INS_1) that affect the coding regions of *CTSH*, *SCAPER*, and *REXO5*, respectively (Table S3).

#### Top Candidate Gene Selection.

Analysis of SNVs/indels, CNVs, and SVs within the linkage regions yielded a total of 75 genes associated with LI*, and 36 genes associated with RI*. Genes were then selected as top candidates based on the following criteria: 1) brain expression median TPM value > 5 in at least 1 database and 2) gene contain variants that passed segregation filtering in multiple families. After this filtration, there were 34 candidate genes associated with LI* (Table S4a), and 13 candidate genes associated with RI* (Table S4b). To select the top candidate genes, genes were further analyzed based on segregation patterns, previous literature, and metrics such as pLI and LOUEF. Genes with an established association with NDDs were prioritized. Ten genes were identified as the top candidates for LI*, and six genes were identified as the top candidates for RI* (Table 3). The variant and gene counts from each step are summarized in Figure 3. Specific genes of interest associated with each phenotype are further discussed below.

#### LI* Genes of Interest.

In the final LI* candidate gene set, the highest-ranking gene is Zinc Finger Protein 774 (*ZNF44*). On the variant level, one non-synonymous SNV and one CNV are found within *ZNF774*. The non-synonymous variant (15–90904468-C-T) segregates in one family with two affected members and has a gene LOD score of 0.46. The CNV, 15_90794757_90950358_<CN3>_1, segregates within another family. The CNV is a 156kb duplication that affects 6 genes, including *ZNF774*. *ZNF774* has a brain expression median TPM value of > 5 in two databases, GTEx and HDBR. Specifically, *ZNF774* has strong expression in the basal ganglion, diencephalon, and medulla oblongata during multiple developmental stages. In particular, the basal ganglion has been previously implicated in autism, and plays an essential role in motor skill acquisition and development (Prat et al. 2016). Alterations to the basal ganglion disrupts the normal flow of feedback to the cortex, thus impacting basic functions such as higher order cognition, gross and fine motor skills, and speech (Subramanian et al. 2017). The medulla oblongata has also been shown to be associated with autism – one study found that the medulla oblongata is significantly smaller in autistic children than in control children (Hashimoto et al. 1993). *ZNF774* is categorized as a C2H2 zinc finger protein (C2H2-ZNF), which act as important targets for pathologic processes associated with NDDs, and are highly expressed in the developing brain. C2H2-ZNFs play a significant role in the regulation of brain morphogenesis and influence the proliferation and migration of neural stem cells. In particular, *ZNF774* has been found to be one of the C2H2-ZNFs implicated in the pathogenesis and pathophysiology of ASDs and autistic features (Mackeh et al. 2018).

Another gene of interest within the LI* linkage region is S-Phase Cyclin A Associated Protein in the ER (*SCAPER*). *SCAPER* has a non-synonymous variant (15–76726530-C-T) segregating in one family with two affected members and has a LOD score of 0.46. SCAPER also has one SV, 15_77092501_77092758_INS_1, which segregates in two families. This SV overlaps the coding region between intron 7 and intron 8 and it is predicted to be a frameshifting mutation. *SCAPER* has a brain expression median TPM value of > 5 in all three databases, strongly indicating expression in the brain. *SCAPER* is shown to have high expression in the pre-frontal cortex across various developmental stages. The pre-frontal cortex has been frequently implicated in autism, and is primarily responsible for deficits in higher-order functions such as cognition, language, and emotion (Rinaldi, Perrodin, and Markram 2008). Additionally, *SCAPER* has been shown to be associated with intellectual developmental disorder and speech disorder (Tatour et al. 2017).

#### RI* Genes of Interest.

In the final RI* candidate gene set, the highest-ranking gene is Xylosyltransferase 1 (*XYLT1*). *XYLT1* has 3 non-synonymous SNVs segregating within 4 families with an overall pVAAST LOD score of 1.59, which implies a strong segregation of variants within families. *XYLT1* also has one SV, 16_17414680_17414960_INS_1, which is an intronic variant that segregates in one family. *XYLT1* has a brain expression max TPM value of > 5 in all three databases. Specifically, *XYLT1* is highly expressed in the amygdaloid complex, which has been previously implicated in autism and other NDDs. Studies have found that the amygdala is an important component of the neural network responsible for social cognition and brain function. Impairment in the amygdala has been shown to cause abnormal social behavior, as well as various NDDs (Amaral and Corbett 2003). Additionally, autistic children have been shown to have significantly slower right amygdala growth (Andrews et al. 2022). The high expression of *XYLT1* in the amygdaloid complex suggests that variants within the gene may contribute to these NDDs. Variants in *XYLT1* have also been shown to contribute to disorders associated with developmental delay, such as Baratela-Scott syndrome (BSS) (LaCroix et al. 2019).

Another gene of interest in the RI* linkage region is the Nonsense Mediated MRNA Decay Associated PI3K Related Kinase (*SMG1*). *SMG1* has an overall pVAAST LOD score of 0.61, implying relatively strong segregation of variants within families. One missense variant (16–18907410-A-G) segregates within one family with one affected member. The other missense variant (16–18908268-C-T) segregates in another family with 2 affected members. *SMG1* has one candidate SV, 16_18832544_18838377_DEL_1, an intronic variant that segregates within 7 families, implying strong segregation in affected members. *SMG1* has a brain expression median TPM value > 5 in all three databases. In particular, *SMG1* has relatively high expression in the amygdaloid complex and pre-frontal cortex. As aforementioned, alteration in these brain regions have strong association with autism and other NDDs, therefore implying a potential role of *SMG1* in the developmental of these phenotypes. Additionally, *SMG1* has been previously identified as an ASD candidate gene, with an essential role in the Nonsense-Mediated mRNA Decay (NMD) pathway (Marques et al. 2022).

## DISCUSSION

This research aimed to delineate candidate genes in the previously identified linkage peaks for language and reading on chromosomes 15 and 16. It refined the implicated regions by adding more families to the analysis, reducing the critical regions for analyzing variants in those regions. We further sought to assess the generalization of these findings across two family ascertainment criteria.

To identify candidate risk genes within the linkage peaks for LI* and RI*, we examined different types of genomic variants, including SNVs, indels, SVs, and CNVs. Through analysis of the variant sets, we determined a set of 35 genes associated with LI*, and 14 genes associated with RI*. Within each phenotype, there were genes with variants that segregate within multiple families, as well as previously established associations with other NDDs. Additionally, several genes are highly expressed in brain tissues associated with neurodevelopmental processes. We applied a set of criteria to further prioritize genes based on factors such as brain expression and segregation patterns, as well as an extensive literature search. Our final results include 10 top candidate genes for LI*, and 6 top candidate genes for RI* within the linkage regions (Table 3). In addition to genes previously implicated in NDDs (e.g., *ZNF774* and *DNAH3*), we also identified genes that were not previously associated with NDDs but showed strong evidence of being involved in ASD and language impairment in our cohort. These discoveries provide new insights into the genetic etiology of language-related traits. The second wave of NJLAGS recruitment (WAVE2) was designed to address the question of how genetic heterogeneity in ASD and LI is manifested through phenotypic heterogeneity, in this case, driven by two sets of ascertainment criteria, one strict (WAVE1) and one more relaxed (WAVE2). Interpretation of the results is based on the dichotomy that either the magnitude of the genetic findings is increased or decreased by the inclusion of WAVE2. In the results, both trends occurred, indicating that this design can be informative regarding genetic heterogeneity. At a finer scale of thought, we note that our design concerns phenotypic components of ASD rather than ASD itself. Since we did not require multiplex ASD families as part of our ascertainment scheme, our findings may not relate to ASD susceptibility as a unitary construct, the way affected sib-pair for linkage or trios (or case-control) for association do. Instead, based on our requirement of both ASD and LI probands, our findings relate to 1) the relationship between ASD and LI and 2) dimensional aspects of ASD manifested by quantitative instruments such as the SRS. To this end, the NJLAGS study offers unique information regarding ASD.

Based on the main research question, we found that the WAVE2 sample provides no evidence for linkage to LI* on chromosome 15 or RI* on chromosome 16. Both of these loci relate ASD in addition to oral and written language, respectively. However, the relationship only holds for strict ASD and strict LI ascertainment (i.e., WAVE1). WAVE2 included cases of ASD and LI that are of clinical interest but are further from the classic definitions of their respective diagnostic categories. Specifically, the WAVE1 ASD probands had a primary diagnosis of AD, but the majority were also non-verbal or minimally verbal, which emphasizes the potential overlap of language impairment with ASD as well as the fact that in WAVE1, the social and repetitive behaviors were more severe. The WAVE2 LI diagnosis (removal of the IQ criteria) might not be expected to affect the linkage results appreciably. Indeed, while clinicians may see more patients typical in WAVE2 ascertainment, our results indicate that strict clinical definitions may indicate a more genetically homogeneous group to consider—at least in regard to how language relates to ASD in those patients.

For the SRS we expected homogeneity since quantitative traits are not typically modeled with heterogeneity parameters. However, in our samples, the SRS shows heterogeneity across WAVE1 and WAVE2. WAVE2 strengthen the two previous WAVE1 SRS peaks on 14q (for the categorical trait) and 15q (for the quantitative trait) (Bartlett et al. 2014). The peak on 14q went from a PPL of 37% to a PPL of 55% and decreased the critical region by 1 Mb. The peak on 15q went from a PPL of 52% to a PPL of 93% in the present study. In joint analysis of WAVE1 and WAVE2, 15q also showed a PPL of 48% for the categorical SRS trait, which had contributions from both waves. Taken together, these results show a consistency in the genetics of strictly and broadly defined ASD. However, when looking at chromosomes 3 (WAVE 1 only), as well as 19 and 20 (both WAVE2 only), the linkage findings were clearly discordant across WAVE1 and WAVE2. This level of heterogeneity was not expected for a quantitative trait. It may be caused by differences in ascertainment that effectively create different underlying populations for quantitative trait analysis.

The difference in how ASD relates to LI versus how ASD relates to general social constructs may have implications for meta-analysis methods. Based on our data, we might posit that meta-analysis of the SRS could be highly productive at some but not all loci. However, combining ASD samples for a joint analysis of language traits would require a model that incorporates differences in ASD ascertainment and LI ascertainment. As our study has strict ASD confounded with strict LI, we cannot know if the strictness of ASD and/or the strictness of language impairment criteria are responsible for this result. Yet one or both would need to be included in meta-analysis models to retain true results, rather than wash out true effects due to the genetic heterogeneity across the studies included.

We chose to examine and report the SRS Restricted Interests and Repetitive Behavior Treatment subscale in this follow-up study—a departure from our use of the Yale-Brown Obsessive Compulsive Scale (YBOCS) in the original study—for two reasons. 1) As mentioned previously, when the study first began in 2003, and the initial analysis period in 2010, adult T-scores were not available for the SRS even though specific behaviors were available as a sub-category (“autistic traits”). At that time, the YBOCS offered a scale of behaviors that best represented a range of OCD-like behaviors comparable to restricted interests and repetitive behaviors (RIRB) characterizing ASD. 2) Once the SRS-2 was published, it offered a standardized (RRB) subscore and contained questions specifically addressing behaviors related to ASD. While the YBOCS focuses on OCD behaviors specific to that disorder, administration requires a degree of interpretation both by the clinician and the respondent. The decision to use the SRS-RIRB was based on issues with YBOCS coding and making it a quantitative trait for genetic analysis. It was not designed for that purpose, while the SRS was. However, one aspect in favor of the YBOCS is that it fundamentally measures repetitive behaviors and obsessive-compulsive disorder, while the SRS is more social in its design space. Yet, the two may be much more closely aligned, which is sometimes appreciated. A recent paper by Gulisano and colleagues (Gulisano et al. 2020), examined the overlap of OCD-like behaviors in children initially diagnosed with Tourette Syndrome using the YBOCS while also administering the SRS-2. They suggested that some of the OCD-like behaviors associated with Tourette Syndrome might be confused with stereotypies associated with ASD.

Every peak contained at least one SFARI (Abrahams et al. 2013; Banerjee-Basu and Packer 2010) autism candidate gene (Table 4). Our study implicated ~ 3.8% of the human genome and the list of SFARI genes implicates ~ 3.7% of the known human genes. If our observed linkage signals were random noise, we would expect very low concordance between the two. The fact that so many SFARI genes occur in our findings indicates a convergence of methodologies. Our ascertainment scheme differs from typical ASD research, yet the genetic findings overlap quite compellingly. This finding both validates our approach and adds further evidence for the genes listed in Table 4 as having a role in ASD.

Our candidate gene study has some limitations. One is that the candidate risk genes’ functional impact is determined primarily through annotations. While gene annotations provide a detailed description of the functional impact, the true impact on the phenotype of interest usually needs additional confirmation. Therefore, one potential expansion of this study is to focus on functionally validating the candidate genes through experiments, such as testing the genes/variants in human iPSCs or mice. Another limitation is the relatively small sample size of the NJLAGS cohort. Large SVs and CNVs are primarily impacted by this small sample size, as some variants may falsely be given higher prioritization. Because of the highly heterogeneous nature of ASD, a future study with a larger sample size can further increase the statistical power for candidate gene prioritization. Additionally, a whole genome analysis beyond the linkage regions can be conducted to examine variants and genes in other parts of the genome, and gather a more comprehensive understanding of the genetic architecture of ASD and language impairments.

In conclusion, our analysis of the NJLAGS cohort provides further elucidation of the genetic architecture and interaction of ASD and language-related phenotypes. In addition, we reported a number of high-confidence candidate genes within the linkage regions on chromosomes 15 and 16. In the future, these genes will require functional validation to define their role in neurodevelopment.

## Figures and Tables

**TABLE 1.** Linkage Analysis Results of Autism Spectrum Disorder and Specific Language Impairment

							Maximizing Model				
							Estimated Genotypic Effect for Locus^c^				
Phenotype^a^	Chr	cM	PPL	Band Range	Width (Mb)	Fully Maximized Lod Score^b^	−/−	−/+	+/+	Disease Gene Frequency	Heterogeneity Parameter in Admixture
											Likelihood (a)^d^
LI*	15	84 (76–113)	***0.57***	15q23–26	23.8	4.01	0.0	0.7	0.9	0.001	0.9
RI*	16	45 (39–51)	***0.33***	16p12.1–3	7.3	3.93	0.0	0.0	0.8	0.8	1.0
SRS-TS	2	70 (69–82)	***0.41***	2p16–21	14.6	4.21 (W1)	1.0	3.0	3.0	0.01	1.0
SRS-TS	3	23	***0.34***	3p25.3	3.9	4.45 (W1)	−3.0	−3.0	−1.0	0.01	0.8
SRS-TS	15	129 (114–133)	***0.93***	15q23–26	23.9	4.38 (W1)	−1.0	2.0	3.0	0.01	0.7
SRS-TS	19	17 (1–25)	0.27	19p13.2–3	8.1	3.48 (W2)	−1.0	−1.0	1.0	0.01	1.0
SRS-TS	20	100 (97–103)	***0.36***	19q32–33	1.3	2.46 (W1)	2.0	2.0	3.0	0.99	0.8
SRS-TS-DT	14	115 (106–122)	***0.55***	14q32.2–3	6.7	3.27 (W1)	0.0	0.4	0.99	0.01	0.95
SRS-TS-DT	15	129 (124–133)	***0.48***	15q26	2.7	2.33 (W1)	0.0	0.4	0.99	0.1	1.0
SRS-RIRB	3	15 (10–28)	***0.71***	3p25–26	7.3	4.23 (W12)	−3.0	−3.0	−2.0	0.01	1.0
SRS-RIRB	19	24 (1–25)	**0.42**	19p13.2–3	7.8	3.58 (W2)	−3.0	3.0	3.0	0.99	1.0

a LI* represents oral language impairment and/or ASD. RI* represents reading impairment and/or ASD.

b Sometimes referred to as a Mod score.

c For categorical analysis these quantities are penetrances, and for quantitative traits they are genotypic means on a z-scorescale.

d Estimate of the proportion of families linked to a given locus.

SRS-TS: SRS T-score; SRS-TS-DT: SRS T-score dichotomous trait; SRS-RIBR: SRS Restricted Interests and Repetitive Behaviors.

**TABLE 2.** Breakdown of Linkage Results by Wave and Tier

Phenotype	Chr	Sequentially updated Tiers and Waves^1^	Pooled Tiers and Waves	WAVE1^2^	WAVE2^2^	Pooled Tiers-Sequentially updated Waves
LI*	15	0.57	0.44	0.56	0.02	0.61
RI*	16	0.33	0.23	0.34	0.019^3^	0.32
SRS-TS	2	0.41	0.13	0.21	0.06	0.46
SRS-TS	3	0.34	0.26	0.51	0.012^3^	0.50
SRS-TS	15	0.93	0.80	0.83	0.05	0.80
SRS-TS	19	0.27	0.06	0.0098^3^	0.51	0.17
SRS-TS	20	0.36	0.11	0.012^3^	0.52	0.11
SRS-TS-DT	14	0.55	0.21	0.54	0.022^3^	0.38
SRS-TS-DT	15	0.48	0.10	0.33	0.04	0.10
SRS-RIBR	3	0.71	0.25	0.78	0.015^3^	0.27
SRS-RIBR	19	0.42	0.11	0.04	0.41	0.32

1 This is the primary analysis model presented in this publication.

2 Results from updating over tier within a single wave (to two significant digits).

3 PPL’s below 2% are evidence against linkage (and by extension, neutral evidence=2% while positive evidence > 2%).

SRS-TS: SRS T-score; SRS-TS-DT: SRS T-score dichotomous trait; SRS-RIBR: SRS Restricted Interests and Repetitive Behaviors.

**TABLE 3.** Top candidate genes for LI* and RI*

Gene	Gene Name	Variant Type	Variant	Function	Segregating Families	Known NDD
*SCAPER*	S-phase cyclin A-associated protein in the ER	SNV	15-76726530-C-T	non-synonymous	1	0
		SV	15_77092501_77092758_INS_1	CDS	2	
*CPEB1*	cytoplasmic polyadenylation element binding protein 1	SNV	15-83240107-G-T	non-synonymous	1	0
		SNV	15-83296111-G-T	non-synonymous	1	
*ADAMTSL3*	ADAMTS-like 3	SNV	15-84506964-A-G	non-synonymous	2	0
		SNV	15-84651321-C-T	non-synonymous	1	
		SV	15_84643753_84644082_DEL_1	intronic	1	
*AKAP13*	A kinase (PRKA) anchor protein 13	SNV	15-86198852-C-T	non-synonymous	1	0
		SV	15_86224963_86225242_INS_1	intronic	2	
*ZNF774*	zinc finger protein 774	SNV	15-90904468-C-T	non-synonymous	1	1
		CNV	15_90794757_90950358_<CN3>_1	CN3	1	
*WHAMM*	WAS protein homolog associated with actin golgi membranes and microtubules	SNV	15-83478990-C-T	non-synonymous	1	0
		SNV	15-83499472-C-T	non-synonymous	1	
*KIAA1024*	KIAA1024	SNV	15-79750056-C-T	non-synonymous	1	0
		SNV	15-79750609-A-C	non-synonymous	1	
*ACAN*	aggrecan	SNV	15-89389050-C-T	non-synonymous	1	0
		SNV	15-89400204-C-T	non-synonymous	1	
		SNV	15-89401134-C-T	non-synonymous	1	
		SNV	15-89401362-G-T	non-synonymous	1	
*CSPG4*	chondroitin sulfate proteoglycan 4	SNV	15-75975211-C-T	non-synonymous	1	0
		SNV	15-75980780-A-G	non-synonymous	1	
		SNV	15-75982312-C-T	non-synonymous	2	
*THSD4*	thrombospondin type 1 domain containing 4	SV	15_71885481_71885814_DEL_1	intronic	7	0
		SV	15_71991555_71991778_DEL_1	intronic	5	
*XYLT1*	xylosyltransferase I	SNV	16-17211545-C-T	non-synonymous	1	0
		SNV	16-17232234-A-G	non-synonymous	2	
		SNV	16-17353170-C-G	non-synonymous	1	
		SV	16_17414680_17414960_INS_1	intronic	1	
*SMG1*	smg-1 homolog phosphatidylinositol 3-kinase-related kinase (C. elegans)	SNV	16-18907410-A-G	non-synonymous	1	0
		SNV	16-18908268-C-T	non-synonymous	1	
		SV	16_18832544_18838377_DEL_1	intronic	7	
*ARHGAP17*	Rho GTPase activating protein 17	SNV	16-24950845-C-T	non-synonymous	1	0
		SNV	16-24950880-C-T	non-synonymous	1	
*USP31*	ubiquitin specific peptidase 31	SNV	16-23080170-A-G	non-synonymous	1	0
		SNV	16-23160057-C-T	non-synonymous	1	
*DNAH3*	dynein axonemal heavy chain 3	SNV	16-20994160-A-G	non-synonymous	1	1
		SNV	16-21008629-C-T	non-synonymous	1	
		SNV	16-21031053-C-T	non-synonymous	1	
*TNRC6A*	trinucleotide repeat containing 6A	SNV	16-24800831-A-G	non-synonymous	1	0
		SNV	16-24828224-A-G	non-synonymous	1	

**TABLE 4.** Overlap of current findings with SFARI ASD gene list

Trait	Chromosome	SFARI Genes
SRS-Total Score	2	FBXO11, NRXN1
SRS-Repetitive	3	CX3CR1, CTNNB1, SETD2, DHX30, PLXNB1, QRICH1, GPX1, AMT, ACY1, CACNA1D, CACNA2D3, ASB14, FHIT, FEZF2, CADPS, PRICKLE2, FOXP1, CNTN3, ROBO2, CADM2, TBC1D23
SRS-Total Score	3	FOXP1, CNTN3 , ROBO2, CADM2
SRS-Total Score Categorical	14	CCNK, YY1, DYNC1H1, CDC42BPB
LI*	15	CELF6, BBS4, NEO1, CD276, SIN3A, CIB2, ARNT2, NTRK3, ZNF774, ST8SIA2, CHD2
SRS-Total Score Categorical	15	ALDH1A3
SRS-Total Score	15	ALDH1A3
RI*	16	SYT17, DNAH3, PRKCB
SRS-Total Score	19	KDM4B
SRS-Total Score	20	GNAS

## Data Availability

The raw sequencing reads, variants, and genotypes for all samples are available in the National Institute of Mental Health (NIMH) Data Archive (NDA) under collections C1932 and C2933 and NRGR under study 39. Access can be requested through the portal at this link (https://nda.nih.gov).

## AVAILABILITY OF DATA AND MATERIALS

The code and work flow for the project are publicly available on GitHub:
